# The Flavivirus Non-Structural Protein 5 (NS5): Structure, Functions, and Targeting for Development of Vaccines and Therapeutics

**DOI:** 10.3390/vaccines12080865

**Published:** 2024-08-01

**Authors:** Jarvis Z. H. Goh, Lachlan De Hayr, Alexander A. Khromykh, Andrii Slonchak

**Affiliations:** Australian Infectious Diseases Research Center, School of Chemistry and Molecular Biosciences, The University of Queensland, St. Lucia, QLD 4072, Australia; jarvis.goh@uq.edu.au (J.Z.H.G.); l.dehayr@uq.edu.au (L.D.H.); alexander.khromykh@uq.edu.au (A.A.K.)

**Keywords:** flavivirus, NS5, vaccine, Zika virus, dengue virus, West Nile virus, RdRP, antivirals

## Abstract

Flaviviruses, including dengue (DENV), Zika (ZIKV), West Nile (WNV), Japanese encephalitis (JEV), yellow fever (YFV), and tick-borne encephalitis (TBEV) viruses, pose a significant global emerging threat. With their potential to cause widespread outbreaks and severe health complications, the development of effective vaccines and antiviral therapeutics is imperative. The flaviviral non-structural protein 5 (NS5) is a highly conserved and multifunctional protein that is crucial for viral replication, and the NS5 protein of many flaviviruses has been shown to be a potent inhibitor of interferon (IFN) signalling. In this review, we discuss the functions of NS5, diverse NS5-mediated strategies adopted by flaviviruses to evade the host antiviral response, and how NS5 can be a target for the development of vaccines and antiviral therapeutics.

## 1. Introduction

Mosquito- and tick-borne flaviviruses include some of the most important human pathogens, such as dengue (DENV1–4), Zika (ZIKV), West Nile (WNV), yellow fever (YFV), Japanese encephalitis (JEV), and tick-borne encephalitis viruses (TBEV) [1]. Their primary vectors are mosquitoes belonging to the Culicidae family [2]. *Aedes aegypti* (*Ae. aegypti*) and *albopictus* (*Ae. albopictus*) are responsible for DENV, ZIKV, and YFV transmission, whereas Culex sp. is responsible for WNV and JEV transmission [2]. TBEV is primarily transmitted by ticks in the Ixodidae family, with the three species of greatest concern being *Ixodes ricinus*, *Ixodes persulcatus,* and *Haemaphysalis concinna* [3,4]. Once introduced into the skin through a mosquito or tick bite, flaviviruses are highly capable of disseminating into circulation and infecting multiple organs and cell types [2]. Flavivirus diseases display a wide spectrum of clinical manifestations, with most cases ranging from asymptomatic to mild fever and self-limiting [1]. In severe cases, infected individuals may develop neurotropic (e.g., encephalitis, Guillain–Barré syndrome) disease exemplified by WNV, ZIKV, JEV, and TBEV infections or visceral (e.g., haemorrhagic fever, hepatitis, or shock syndrome) disease exemplified in DENV infections [1]. Infection with ZIKV has also been linked to the occurrence of fetal microcephaly and other developmental abnormalities [5].

These arthropod-borne pathogens pose a significant disease burden, with DENV alone infecting up to ~400 million individuals each year, causing ~100 million cases with clinical manifestations [6]. In the last 20 years, WNV has been estimated to have caused up to 7 million infections and more than 2300 deaths in the United States [7]. In the Asia-Pacific region, JEV is responsible for ~100,000 cases and 2500 deaths annually [8]. Alarmingly, the threat of flaviviral disease is growing, with conservative projections estimating a five-fold increase in the number of people at risk by 2040–2060, depending on the flavivirus, region, and climate scenario [9,10]. This underscores the urgent need for safe and efficacious vaccines or antiviral therapies for flaviviruses. Currently, there are vaccines available for YFV, JEV, TBEV, and DENV, whereas vaccines for ZIKV and WNV are in various phases of clinical trials (Table 1). The development of novel flavivirus vaccines has remained challenging due to the complex and diverse immunopathology of flaviviruses. Furthermore, no specific treatment exists for flavivirus-induced diseases, largely due to our limited understanding of the mechanisms of their pathogenesis [11]. The non-structural protein 5 (NS5) performs crucial roles in viral replication and the antagonism of host innate immune responses, presenting a multitude of opportunities for the development of novel vaccines or antivirals targeting its functions. In this review, we summarize the various functions of NS5 and its diverse strategies in the antagonism of the innate immune response. Furthermore, we will discuss the potential of NS5 as a vaccine candidate or drug target and propose strategies to exploit NS5 for novel therapeutic interventions.

## 2. Molecular Biology of Flaviviruses

### 2.1. Flavivirus Genome Organisation and Replication Cycle

**Figure 1:**
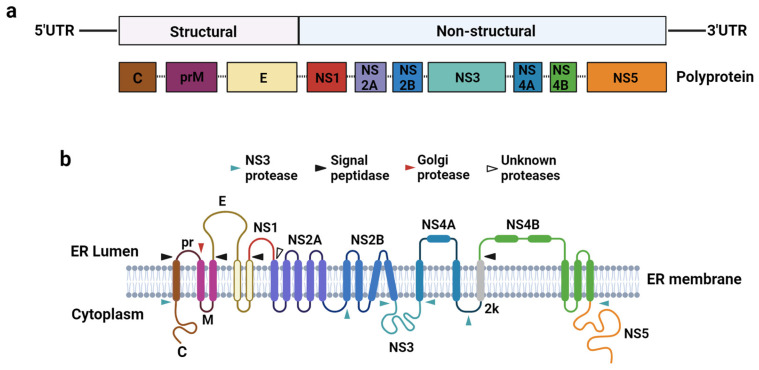
Flavivirus genome organization and polyprotein processing. (**a**) The flavivirus +ssRNA genome is flanked by 5′ and 3′UTRs and encodes for a single ORF that is translated into a single polyprotein in the ER. (**b**) Cleavage of the polyprotein by host and viral proteases generates 10 functional proteins: the 3 structural proteins (C, prM, and E) and 7 non-structural proteins (NS1–NS5). Arrows indicate proteolytic cleavage sites.

Flaviviruses comprise positive-sense single-stranded (+ssRNA) genomes ~11 kb in length containing a Type 1 cap (m7GpppN) at the 5′ terminus but lacking a 3′ polyadenine tail [12] (Figure 1a). The coding region is flanked by the 5′ and 3′ untranslated regions (5′UTR and 3′UTR), with the 5′UTR spanning approximately 100 nucleotides (nt) and the 3′UTR ranging between 400 and 700 nt [13]. The viral-coding region contains a single open reading frame (ORF) that encodes for a single polyprotein that is proteolytically cleaved co- and post-translationally by cellular and viral proteases into three structural proteins: capsid (C), pre-membrane/membrane (prM/M), and envelope (E), and seven non-structural (NS) proteins: NS1, NS2A, NS2B, NS3, NS4A, NS4B, and NS5 [13] (Figure 1b). The structural proteins constitute the components of the assembled pre-mature and mature virions, whereas the non-structural proteins perform various crucial functions during viral replication and contribute to flaviviral pathogenesis.

The flaviviral 5′ and 3′ UTRs contain multiple RNA elements that are essential for viral replication and pathogenesis, such as the 5′ stem loops A and B (SLA and SLB), 5′ and 3′ upstream AUG region (UAR) and conserved/cyclization sequences (5′CS and 3′CS), capsid-coding region hairpin element (cHP), 3′ short hairpin (sHP), and 3′ stem loop (3′SL) [14,15]. Genome cyclization is a critical process involving long-range RNA-RNA interactions and extensive hybridization of the complementary 5′-3′ UARs and CS regions to form a panhandle-like structure [16]. The 5′SLA is recognized by the viral polymerase to initiate negative-sense RNA synthesis [17]. The terminal 3′SL bears a highly conserved pentanucleotide (5′-CACAG-3′) in the apical loop that may facilitate -ssRNA synthesis by recruiting the 5′SLA-bound viral polymerase in proximity to the 3′ end [18]. The 3′SL may also function as a metastable regulator of genome cyclization through the structural flexibility of its stem structure to allow hybridization of the 5′ and 3′ UAR sequences [19]. Furthermore, the 3′UTR contains highly conserved RNA structures that resist exoribonuclease 1 (XRN1) degradation to generate functional non-coding RNAs, known as Subgenomic Flaviviral RNAs (sfRNAs) [20].

Flaviviruses are transmitted through mosquito or tick bites, during which infective virions are deposited into the epidermis and encounter cells that are permissive to infection, such as keratinocytes and skin dendritic cells [21]. Infected dendritic cells migrate to the lymphoid organs, where they infect other immune cells, such as macrophages and monocytes [21]. The ensuing rapid viral replication facilitates the dissemination of flaviviruses into circulation and internal organs [21].

The flaviviral replication cycle is initiated upon the interaction of the E glycoprotein with cell surface receptors for viral adhesion, including glycosaminoglycans (GAGs), which are prominently expressed on cell surfaces [22]. GAGs function as attachment factors to concentrate flaviviral particles at the target cell surface prior to their interaction with primary receptors [22]. Several primary receptors crucial for flavivirus entry have been identified, including αvß3 integrin, C-type lectin receptors, and phosphatidylserine receptors (TIM, TAM: TYRO3, AXL, and MER) [22]. Upon receptor-mediated binding, flaviviruses are internalized by clathrin-mediated endocytosis and transported through an endosomal and lysosomal endocytic pathway, where the progressively decreasing pH environment triggers viral fusion with the membrane of the endocytic vesicle, nucleocapsid disassembly, and release of the +ssRNA genome into the cytoplasm [23].

Flaviviruses then rely on host cell ribosomal machinery for the translation of the +ssRNA through both cap-dependent and -independent initiation mechanisms [23,24]. Eukaryotic initiation factors (eIFs) recognize and bind to the 5′ cap structure to promote recruitment of the 40S ribosomal subunit and associated factors to the viral RNA [25]. Although the translation of the viral RNA can be initiated in the cytosol, it is likely that the flaviviral genome is recruited to the endoplasmic reticulum (ER) and translated under ER-associated ribosomes [25]. The translated viral polyprotein is then cleaved by both cellular and viral proteases into the constituent viral proteins [25]. As the translation and local concentration of viral non-structural proteins increases, these proteins drive the invagination of the ER membrane and induce drastic ER rearrangements in order to form membrane-associated replication complexes (RCs), where viral RNA synthesis occurs [23].

The final stages of the flaviviral life cycle involve the assembly of viral RNA and structural proteins into virion particles, followed by their maturation and release through exocytosis [23]. Virion assembly occurs at the ER, where viral RNA self-assembles with the structural proteins (C, prM, and E) and bud into the ER lumen [25]. Immature virions that accumulate in the ER are transported to the Golgi apparatus in individual vesicles for glycan maturation [26]. The transit of virions through the acidic compartment of the trans-Golgi network promotes a pH-dependent reorganization of the viral surface by enabling furin-mediated cleavage of prM to render the virions mature and infective [26].

During viral replication, all flaviviruses tested to date have demonstrated the ability to subvert the host cellular mRNA decay pathway to generate subgenomic flaviviral RNAs (sfRNAs) [20]. The generation of sfRNA has been shown to be crucial for flaviviral replication, antagonism of host immune responses, and viral pathogenesis (reviewed in [27]). Flaviviruses deficient in sfRNA generation display severe attenuation, cytopathicity, and increased sensitivity to interferon treatment, both in vitro and in vivo [28,29]. The generation of sfRNA by WNV was shown to be crucial for neuropathogenicity in mice models [20]. The expression of sfRNA by ZIKV was also crucial for neurovirulence, as sfRNA-deficient viruses displayed decreased cytopathic effects upon the infection of human cerebral organoids and neural progenitor cells and decreased caspase-3/7-dependent apoptosis [28]. Experiments with pregnant mice models also demonstrate the importance of ZIKV sfRNA for efficient infection of the placenta, transplacental viral migration, and infection of the fetal brain [28]. Recent gene interaction network analysis highlighted a novel link between the pro-apoptotic activity of sfRNA and dysregulated brain development during ZIKV infections via crosstalk with the Wnt-signalling pathway [30]. Collectively, these studies demonstrate the importance of sfRNA for flaviviral replication and pathogenicity, thus making it a promising target for live-attenuated vaccine development (reviewed in [31]).

The molecular biology of flaviviruses significantly contributes to their evolutionary success by enabling efficient replication, adaptation to divergent host ranges of invertebrate vectors and vertebrate hosts, and immune evasion. Their positive-sense RNA genome allows for rapid replication and mutation, facilitating swift adaptation to diverse environments and host defences. In addition, their small genomes encode for viral factors such as non-structural (NS) proteins and sfRNA that enable the viral evasion of host antiviral mechanisms. The high mutation rate, coupled with a robust replication mechanism, enables flaviviruses to evolve quickly, overcome host immune responses, and adapt to different vectors and hosts, contributing to their widespread distribution and persistence in various ecological niches.

### 2.2. Innate and Adaptive Immune Responses against Flaviviruses during an Infection

#### 2.2.1. Innate Immune Response to Flaviviral Infections

Upon cell entry, flaviviruses are detected by pattern-recognition receptors (PRRs). The main PRRs that are critical for the recognition of flaviviruses are the Toll-like Receptors (TLRs) and retinoic acid-inducible gene-1 (RIG-I)-like receptors (RLRs) [32]. TLR3, which is expressed in cell types such as neurocytes, immune cells, fibroblasts, and epithelial cells, can recognize the viral RNA during replication, when double-stranded-RNA (dsRNA) intermediates are formed [33,34]. TLR3 activation initiates the TRIF-dependent pathway to activate interferon-regulatory factor 3/7 (IRF3/7) [34]. Activated TLRs trigger signalling cascades that result in phosphorylation and nuclear translocation of transcription factors NF-κB and IRF3/7 [35]. This leads to transcriptional activation of genes that encode for pro-inflammatory cytokines and interferons (IFNs) [33]. Type I IFN is the most ubiquitous cytokine involved in response to flaviviral infections [36], signalled through transmembrane type I IFN receptor (IFNAR), composed of the subunits IFNAR1 and IFNAR2 [37]. IFN binding leads to ligation of both IFNAR subunits and the activation of the receptor-associated protein tyrosine kinases Janus kinase 1 (JAK1) and tyrosine kinase 2 (TYK2) via auto- and trans-phosphorylation [37]. These kinases then phosphorylate the cytoplasmic signal transducer and activator of transcription 1 (STAT1) and 2 (STAT2), leading to their dimerization and binding of IFN regulatory factor 9 (IRF9) to assemble the IFN-stimulated gene factor 3 (ISGF3) complex [37]. This complex is then translocated into the nucleus, where it binds to IFN-stimulated response elements (ISREs) within promoters of ISGs to promote antiviral gene transcription [37]. Alternatively, IFN-α/β can signal through phosphorylated STAT1 homodimers (also designated as the γ-activated factor (GAF)), which binds to gamma-activated sequences (GAS) in the nucleus and promote type II IFN signalling [37].

As flaviviruses encounter a broad spectrum of antiviral immune responses from their hosts, they have evolved sophisticated strategies to suppress and evade host defences. Central to these strategies are the non-structural proteins that inhibit IFN signalling pathways, dampening the host’s ability to mount an effective antiviral response. By leveraging these non-structural proteins, flaviviruses ensure efficient viral replication and transmission despite host defence strategies.

#### 2.2.2. Adaptive Immunity in Response to Flaviviruses

Humoral immunity, which comprises B cells and antibody responses, plays a critical role against flaviviral infections. The generation of neutralizing antibodies that target structural proteins, such as the prM/M and E proteins, protect against flavivirus infections at various stages of viral entry, including interference of viral receptor attachment to host cells [38] or blocking the fusogenic conformational change of the E protein within the endosome to prevent membrane fusion [39]. Alternatively, antibodies may also inhibit flavivirus infections through Fc-dependent effector functions, including antibody-dependent cellular cytotoxicity (ADCC) and cellular phagocytosis (ADCP) or antibody-mediated complement activation and complement-dependent cytotoxicity (CDC). However, antibodies that target structural proteins pose an increased risk of antibody-dependent enhancement (ADE) of infection, which is a phenomenon in which antibodies fail to neutralize virions and instead enhance viral infectivity by facilitating viral entry into target cells. ADE occurs when virions are bound by poorly neutralizing antibodies (binding to epitopes that are not involved in viral entry) or under sub-neutralizing antibody concentrations (binding to epitopes below the neutralization threshold), resulting in virus-antibody immune complexes that are internalized into target cells through a Fcγ receptor (FcγR)-dependent phagocytic mechanism. Consequently, cells bearing FcγR, such as monocytes, macrophages, dendritic cells, and certain granulocytes, are highly permissive to ADE of infection. A novel mechanism of ADE was reported, whereby antibody recognition of the interface of the dimeric E protein results in dissociation of the dimer and exposure of the fusion loop, a process usually only triggered by the acidic pH of the endosome after viral uptake [40]. The premature exposure of the fusion loop can mediate the fusion of the virus to the plasma membrane at a neutral pH and thus increase viral infectivity [40]. In one example, children who received yellow fever chimeric tetravalent dengue vaccine (Dengvaxia) were exposed to a significant risk of severe dengue due to ADE when infected [41]. For these reasons, the design of safe and effective flavivirus vaccines has remained challenging. Studies have since shown that humoral immune responses and neutralizing antibodies do not confer complete protection against flaviviruses, and cellular immunity mediated by CD4+ and CD8+ T-cell responses may be more crucial to protect against flaviviral infections and prevent severe disease [42,43,44]. In the presence of subneutralizing antibodies, CD8+ T-cells can still protect against severe dengue disease [45]. Importantly, studies have reported that T-cell responses in individuals after primary flavivirus infections or vaccination were vastly targeted against NS proteins [46,47,48]. Therefore, flaviviral NS proteins are attractive vaccine targets as they are not presented on the virion’s surface, reducing the risk of inducing antibodies with ADE potential.

### 2.3. Role of Non-Structural (NS) Proteins in Viral Replication and Immune Evasion

Flaviviruses encode seven non-structural (NS) proteins involved in virus replication, pathogenesis, and viral suppression of host immune responses (Table 2). The NS1 protein (~48 kDa) is a multifunctional glycoprotein indispensable for viral replication, particle assembly, and host immune evasion [49]. NS1 exists in multiple oligomeric forms, first translated as a monomer. However, upon glycosylation in the ER or trans-Golgi network, it rapidly forms a dominant dimeric species or further oligomerization to form hexamers [49]. In the flaviviral-replication complex, NS1 colocalizes with non-structural proteins (NS4A and NS4B) and dsRNA to support viral replication [49]. The hexameric NS1 is soluble and is secreted into the extracellular milieu, where it is responsible for the modulation of host immune responses and vascular permeability [50]. NS2A is a ~22 kDa hydrophobic transmembrane protein that has important functions in viral replication and assembly. NS2A is hypothesized to associate with viral RNA through interactions with the 3′UTR, as well as the capsid-prM-E complexes and NS2B-NS3 during virion assembly [51]. NS2B (~14 kDa) is an integral transmembrane protein, serving as a cofactor for the serine protease NS3 [52]. The transmembrane regions of NS2B anchor NS3 to the ER membrane, forming the active NS2B-NS3 protease complex for substrate recognition and catalysis [52]. NS3 is the second largest viral protein at ~70 kDa and is a multifunctional enzyme possessing proteolytic activity for cleavage of the viral polyprotein precursor, RNA-stimulated nucleoside triphosphatase (NTPase), helicase activity to unwind viral double-stranded RNA intermediates for RNA replication, and RNA 5′-triphosphatase (RTPase) activity to dephosphorylate viral 5′ RNA for capping [53]. NS4A is a ~16 kDa transmembrane protein that plays a pivotal role in ER membrane remodelling and thus replication complex formation [54]. Similarly, the transmembrane NS4B peptide (~27 kDa) is contained within the replication complex, interacting with other viral proteins, including NS1, NS2B, and NS4A, pivotal to bringing viral enzymes NS3 and NS5 in close contact with viral RNA [55]. NS4A and NS4B also modulate autophagy and cellular dysregulation to prevent cell death and facilitate persistent viral infection [56]. The NS5 (~100 kDa) protein is responsible for capping and replication of the viral RNA [57]. In addition, NS5 is the most potent virally encoded inhibitor of host interferon (IFN) signalling [58].

Several studies have reported that T-cell responses in individuals after primary flaviviral infections or vaccinations were vastly derived against NS proteins, mainly towards NS1, NS3, or NS5 [46,47,48]. This suggests that vaccines designed to mainly induce neutralizing antibodies against the E protein may not provide sufficient protection and require the incorporation of non-structural proteins to mount a robust T-cell response. This may indicate a possible limitation with vaccines such as the most advanced tetravalent DENV-YFV chimeric vaccine (CYD-TDV), in which DENV NS proteins are absent. The lower-than-expected protection efficiency conferred by CYD-TDV may be due to the lack of T-cell responses against NS proteins [59]. Furthermore, NS proteins present epitopes that are highly conserved across flaviviruses and between serotypes [60,61,62,63]. Khan et al. (2008) identified 42 NS-protein-derived potential T-cell determinants, of which ~77% and ~61% were conserved across DENV serotypes and across flaviviruses, respectively [63]. The incorporation of these T-cell determinants in vaccine candidates may improve vaccine efficiency by inducing robust T-cell responses and cross-protective immunity.

**Table 2:** Flaviviral NS proteins, their subcellular locations, and functions.

Protein	Molecular Weight	Subcellular Location	Function(s)	Reference
NS1	46–55 kDa	Intracellular; cytoplasm; ER lumen;	Replication complex (RC) component TLR or RLR pathway evasion	[50,64,65,66,67]
		Membrane-associated (dimer)	Humoral immune response activation Complement activation Endothelial leakage	
		Secreted (hexamer)		
NS1′	52–53 kDa	Cytoplasm; ER lumen; secreted	Neuroinvasion Substitute NS1 function	[68,69]
NS2A	22 kDa	ER membrane	Virus-induced membranes Suppressor of (IFN-β) transcription	[70,71]
NS2B	~14 kDa	ER membrane	Cofactor required for NS3 protease	[72]
NS3	~70 kDa	Cytoplasm; nucleus	RNA helicase Serine protease RNA triphosphatase Nucleocapsid triphosphatase	[73,74]
NS4A	16 kDa	ER membrane	Membrane remodelling Antagonism of host IFN responses Induction of autophagy	[75]
NS4B	27 kDa	ER membrane	Interaction with other viral proteins, including NS1, NS2B, NS3, and NS4A Antagonism of host IFN responses	[76,77]
NS5	~100 kDa	Cytoplasm; nucleus	Formation of 5′ type I cap RNA-dependent-RNA-polymerase Antagonism of host IFN responses	[78,79,80]

However, the protective effects of the vaccines based solely on NS proteins are limited as NS proteins are not virion components. Localized within the cytosol and nucleus (NS3/5) or replication complexes within the perinuclear region (NS1–5), these proteins cannot be reached by antibodies [73,81,82]. Therefore, the antibodies produced in response to immunisation with intracellular NS proteins will not have a neutralising effect. The flavivirus NS1 protein is an attractive vaccine candidate as it can be both presented on the cell surface as a membrane-associated dimer (mNS1) and secreted into the extracellular milieu as a hexamer (sNS1) and therefore can elicit strong humoral and cellular immune responses [83]. The secreted form of NS1 was highly detected in the serum of infected individuals, with protein levels positively correlating with disease severity and the risk of developing dengue haemorrhagic fever [83]. DENV s-NS1 was shown to induce inflammatory cytokine release in immune cells through a TLR-4-dependent pathway, which contributes to the vascular leakage of endothelial cells [84]. In addition, NS1 may induce vascular leakage through autophagy-mediated endothelial junction disruption [64] or degradation of the endothelial glycocalyx mediated through heparinase activity [50]. Antibodies against NS1 have been shown to prevent NS1-induced vascular endothelium permeability [85], limit flaviviral replication, and provide protection through complement-dependent cytotoxicity [86,87]. However, anti-NS1 antibodies have also been shown to cross-react with host proteins and cause pathological effects such as liver damage, thus raising safety concerns [88,89,90]. The potential for NS1 as a vaccine candidate has since been extensively discussed in previous reviews [91,92]. Conversely, the potential of NS5 as a vaccine candidate remains relatively unexplored and largely undiscussed.

## 3. Non-Structural Protein 5 (NS5) Is a Multi-Functional Protein Required for Viral RNA Replication and Inhibition of the Innate Immune Response

### 3.1. Structure of NS5 Protein and Its Role in Viral Replication

**Figure 2:**
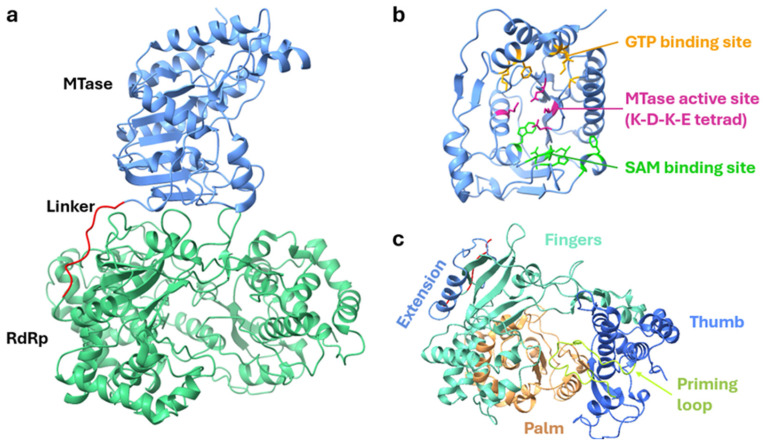
Structure of ZIKV NS5 (PDB, 5U0B). (**a**) Ribbon representation of the MTase and RdRp domains. (**b**) Key residues required for GTP- and SAM-binding and MTase activity. (**c**) The fingers, palm, and thumb subdomains of the RdRp domain; the extension that connects the MTase and RdRp domains through the linker (in red); and the conserved priming loop required for RNA polymerization. For detailed information on the ZIKV MTase and RdRp domains and key residues, refer to [96,97,99,101].

Flaviviral replication occurs within replication complexes, in which viral enzymes and RNA-capping machinery efficiently replicate and protect viral RNA with a 5′ cap modification. Central to flavivirus replication is the non-structural protein 5 (NS5), which is the largest (~100 kDa) and most conserved protein encoded by the flavivirus genome, consisting of two enzymatic domains: the N-terminal Methyltransferase (MTase) and C-terminal RNA-dependent RNA polymerase (RdRp) domains [93] (Figure 2a). The MTase domain (~30 kDa) has dual functions as a guanylyltransferase (GTase) and a methyltransferase. It uses GTP as a substrate to transfer GMP to the NS3-cleaved di-phosphorylated RNA to generate the base cap structure [78]. It then catalyses both guanine N-7 and ribose 2′-OH methylation of the viral RNA using S-Adenosyl-methionine (SAM) as the methyl donor to form the Type 1 Cap structure (m7GpppN) [78]. The guanosine core structure and 2′O methylation protect the 5′ end from recognition by host innate immunity, whereas N-7 methylation is crucial for viral replication by enhancing viral RNA translation [94]. The MTase structure can be further divided into three subdomains. The N-terminal subdomain is characterized by a helix-turn-helix motif, β-strand, and an α-helix, housing the GTP-binding site [95]. The MTase core adopts a canonical Rossmann fold with an α/β/α sandwich structure, in which parallel hydrogen-bonded β-strands (β-strand order of 3–2–1–4–5–7–6) are surrounded by α-helices, also forming the SAM- and GTP-binding sites [96] (Figure 2b). The conserved catalytic K-D-K-E tetrad is also positioned in the centre of the MTase and is crucial for both N-7 and 2′O methylation [97]. The C-terminal subdomain interacts with the N-terminal and core subdomains and consists of an α-helix and a β-strand [95]. The MTase domain is connected via a flexible 10-residue linker to the RdRp domain (~70 kDa). The flexibility of the linker region is crucial for the functions and crosstalk between the MTase and RdRp domains, as mutations incorporated to impose rigidity in the linker result in decreased RdRp activity and the attenuation of viral replication [98]. Like other viral RdRps, the flaviviral NS5 adopts a similar capped ‘right-hand’ structure consisting of three subdomains: palm, fingers, and thumb [96] (Figure 2c). The active site of the RdRp is situated upwards of the palm domain and surrounded by loops from the thumb and palm domains, responsible for the stabilization of RNA molecules during polymerization [99]. Within the active site are two crucial aspartic acids required for binding and positioning two metal ions during nucleotidyl transfer [80]. All flavivirus RdRps studied to date have been shown to initiate RNA synthesis de novo [100]. Accordingly, a priming loop within the thumb domain contains two aromatic residues near the tip (W795 and H798 in DENV RdRp), which are conserved in all flaviviral RdRps [100].

**Figure 3:**
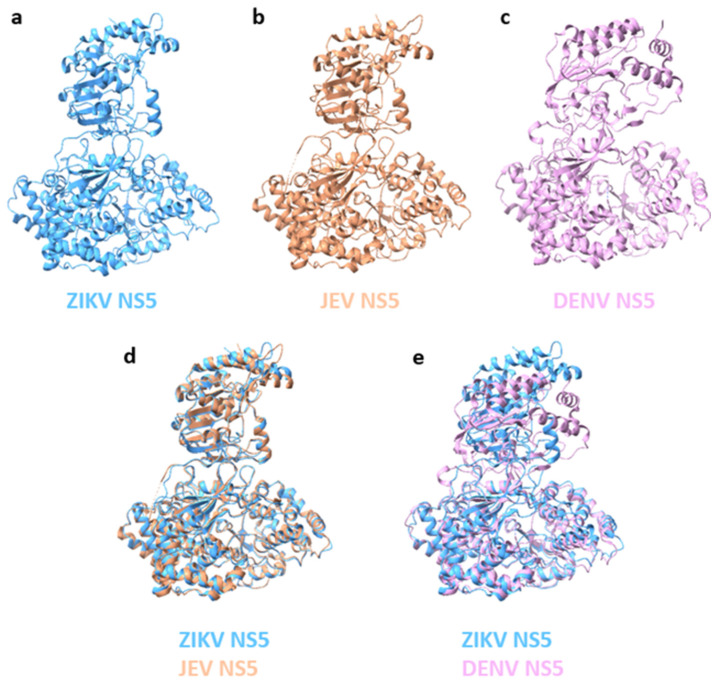
Comparison of ZIKV NS5 structure to JEV and DENV NS5. (**a**) ZIKV NS5 (PDB, 5U0B); (**b**) JEV NS5 (PDB, 4K6M); and (**c**) DENV NS5 (PDB, 4V0Q). (**d**) Superposition of ZIKV and JEV NS5, and (**e**) ZIKV and DENV NS5 structures was performed with ChimeraX (v1.7) software.

The protein architecture of NS5 is well conserved across flaviviruses, as demonstrated by the crystal structures of NS5 from various flaviviruses, including ZIKV, DENV, and JEV [96,101] (Figure 3). However, two alternative domain conformations can be observed: a compact conformation from DENV2 NS5 [102] and an extended conformation from JEV, ZIKV, and YFV NS5 [103,104,105]. Both conformations were observed with DENV2 and ZIKV NS5, suggesting that NS5 can adopt both conformations in a solution [106]. In the compact conformation, the structure of ZIKV and JEV NS5 are highly similar, with the MTase domain located at an acute angle to the RdRp, whereas DENV NS5 in the extended conformation displays a distinct orientation due to a short 310-helix in the linker that rotates the MTase towards the RdRp [96]. The compact conformation of NS5 may facilitate viral RNA replication [107], whereas the extended conformation may function to antagonize interferon signalling through the binding of STAT2 [108].

The NS5 of flaviviruses, including DENV, ZIKV, and JEV, have been shown to assemble into dimers or higher-order oligomeric states [103,109,110]. Small-angle X-ray scattering demonstrated that ZIKV NS5 exists in a monomer–dimer equilibrium in solution [110]. Size-exclusion chromatography (SEC) and Analytical Ultracentrifugation (AUC) experiments performed with recombinant ZIKV NS5 revealed predominant monomeric NS5 (~100 kDa), a secondary species (~10%) corresponding to NS5 dimers (~200 kDa), and less abundant (~5%) species corresponding to higher-order oligomers [111]. NS5 oligomerization was concentration-dependent, with the dimer becoming the dominant form at 1 mg/mL, transitioning to the oligomeric form as the concentration increases [110,112]. The quaternary structure of ZIKV NS5 unveiled two distinct protein arrangements: Type I interactions, where two NS5 molecules bind head-to-tail, and Type II dimer–dimer interactions, together resulting in the formation of large helicoidal fibril-like structures that can be observed under electron microscopy [111].

The function of NS5 oligomerization is unclear, but studies propose that NS5 dimers may coordinate RNA replication within the flaviviral-replication complex [109]. The physical linkage between the domains of NS5 suggests that RNA capping and synthesis are coupled during genome replication; however, analysis of the monomeric NS5 structure revealed that the MTase and RdRp active sites are oppositely facing and do not interact [112]. The highly conserved GTR motif within the N-terminus of the interdomain linker may function as a hinge to facilitate movement in the monomeric form [57]. An alternate hypothesis proposes that in the dimeric form, the RdRp RNA exit site of one monomer and the MTase active site of the partner face the same direction, therefore permitting coordination between the MTase and RdRp domains without requiring large conformational changes [109].

The multiple crystal structures of NS5 have since offered detailed insights into the functional roles of its domains during viral replication, as well as opportunities for the development of novel live attenuated vaccines or antivirals in structure–activity relationship (SAR) studies. The oligomerization of NS5 observed in these studies may have implications in the design of future therapeutics due to the possibility of antivirals having reduced effectiveness against NS5 oligomers.

### 3.2. Flavivirus NS5-Mediated Inhibition of Interferon Signalling

**Figure 4:**
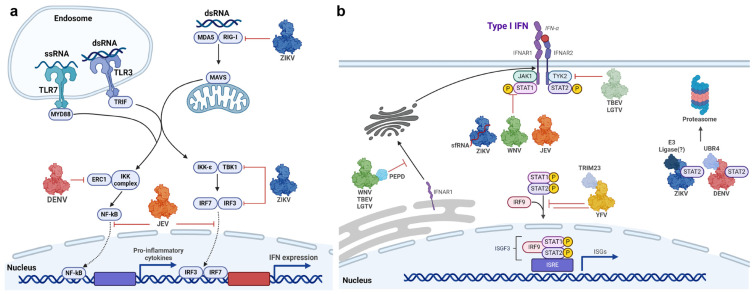
The diverse strategies of flaviviral NS5-mediated inhibition of the innate immune response. (**a**) Viral RNAs are recognized by host–pathogen recognition receptors, TLR3/7 and RIG-I/MDA5, leading to downstream activation of transcription factors NF-κB and IRF3/7. ZIKV NS5 inhibits IFN induction through interference in RIG-I, IKKε, TBK1, and IRF3 signalling. DENV NS5 inhibits the pro-inflammatory activity of NF-κB by inducing ERC1 degradation. JEV inhibits the nuclear translocation of NF-κB and IRF3/7. (**b**) ZIKV, WNV, and JEV NS5 inhibit STAT1 phosphorylation. ZIKV and DENV NS5 subvert host E3 ligases to induce STAT2 degradation. YFV NS5 interacts with STAT2 to inhibit ISGF3 engagement with ISREs. WNV, TBEV, and LGTV disrupt the maturation of IFNAR1 by binding to PEPD. TBEV and LGTV NS5 inhibit JAK-STAT signalling.

Flaviviruses employ multiple strategies to disrupt various steps of the IFN pathway, aiming to delay or prevent the onset of an antiviral response (Figure 4a,b). To date, every flavivirus tested has shown the capacity to counteract IFN signalling, with multiple non-structural proteins implicated in this inhibition [113]. Of them, the NS5 protein has been demonstrated to be the most potent virally encoded antagonist of IFN signalling [58]. Despite the structural and functional constraints of NS5, such as its main role in genome replication, flaviviruses have evolved distinct NS5-mediated mechanisms to antagonize IFN signalling.

DENV NS5 was demonstrated to bind and deplete STAT2 via ubiquitin-dependent proteasomal degradation [114]. Although ectopically expressed NS5 alone can bind and inhibit STAT2 phosphorylation, STAT2 degradation was only observed when NS5 underwent proteolytic processing from the DENV polyprotein. It was hypothesized that the NS5-dependent degradation of STAT2 was regulated under the N-end rule, which involves cleavage of proteins at their N-terminus followed by proteasomal degradation [115]. The first ten N-terminal amino acids of DENV NS5 (especially Thr2 and Gly3, which are conserved amongst all DENV serotypes) were important for binding to ubiquitin-protein ligase E3 component N-recognin 4 (UBR4) [116]. UBR4 is an E3 ubiquitin ligase of the N-recognin family, which targets destabilizing N-terminal residues, an optimal lysine residue (site of polyubiquitylation), and an unstructured N-terminal extension [115]. DENV NS5 binds UBR4 irrespective of whether the N-terminus of NS5 was generated through proteolytic processing, although binding was greater with processed NS5 [116]. The atomic structure of the DENV NS5-human STAT2 (hSTAT2) complex has since been elucidated by cryo-EM, demonstrating that one end of the hSTAT2 CCD domain bound to an interdomain cleft between both the MTase and RdRp domains [79]. Thus, DENV NS5 may utilize its N-terminus and STAT2-binding site to function as a bridge between STAT2 and UBR4. In this role, DENV NS5 may act in self-sacrifice to mediate STAT2 proteasomal degradation but with minimal impact on viral replication, as NS5 is expressed in excess [117]. DENV NS5-mediated STAT2 degradation was not dependent on the nuclear localization of NS5, suggesting that STAT2 binding and degradation occur in the cytosol [118]. DENV NS5 also contains SUMO-interacting motifs (SIM), and SUMO modification of NS5 was found to be crucial for protein stability and effective STAT2 degradation [119]. NS5-UBR4 interaction is also required for proteasomal degradation of ELKS/RAB6-interacting/CAST family member 1 (ERC1) and reducing NF-κB activation in DENV1–3 infections, but not DENV4 [120].

The YFV NS5 employs a unique mechanism to antagonize IFN signalling, as it binds and sequesters, but not degrade, STAT2 only under an IFN-activated state [121]. IFN stimulation induces K63-linked polyubiquitination of NS5 and phosphorylation of STAT1, both important for NS5-STAT2 interaction. It is proposed that YFV NS5 only interacts with STAT2 in the form of STAT1/2 heterodimers, as dimerization induces a STAT2 conformation that permits NS5 binding. Like DENV, the first 10 residues were crucial for YFV NS5 interaction with STAT2, but with the requirement of TRIM23 polyubiquitylation at the K6 residue [121]. YFV NS5 may prevent the association of STAT1/2 with IRF9 to block the formation of ISGF3.

The Japanese encephalitis virus (JEV) NS5 is also a potent IFN antagonist, demonstrated by its ability to suppress the antiviral effects of IFN-α stimulation [122]. JEV NS5 inhibits TYK2 and STAT1 phosphorylation through the subversion of cellular protein tyrosine phosphatases (PTPs) [122]. Additional studies suggest that JEV NS5 downregulates the ER chaperone Calreticulin to inhibit STAT1 nuclear translocation [123]. JEV NS5 was also shown to directly inhibit the nuclear translocation of IRF3 and NF-κB and their transcriptional activities by competitively recruiting importins α3 and α4 [124].

ZIKV NS5 is involved in the disruption of multiple processes within the interferon pathway, including suppressing host recognition, IFN production, and JAK/STAT signalling. ZIKV NS5 was shown to interact with and suppress K63-linked polyubiquitination of RIG-I and inhibit activation and nuclear translocation of IRF3, leading to decreased production of IFN-β [125]. The ability of NS5 to inhibit RIG-I signalling is dependent on the MTase domain and an intact active site, specifically the catalytic K-D-K-E tetrad, but not dependent on MTase activity. ZIKV NS5 also interacts with TBK1 through its ubiquitin-like domain (ULD), which prevents TBK1-TRAF6 interaction. This reduces TBK1 and IRF3 activation to suppress IFN production [126]. In contrast, another study reported the lack of an inhibitory effect of NS5 on TBK-1 but instead demonstrated that NS5 interacted with and inhibited IKK-ε kinase to reduce IRF3 phosphorylation and activation [127]. ZIKV NS5 was also shown to directly interact with IRF3 to suppress IFN-β production [128]. The NS5 of several flaviviruses, including ZIKV, WNV, and JEV, was reported to interact with host heat shock 90 protein (HSP90) [129]. HSP90 is a chaperone involved in regulating the stability of a broad range of proteins, including JAK1/2; inhibition of HSP90 increases proteasomal degradation of JAK1/2 and Tyk2 [129,130]. It is hypothesized that NS5-HSP90 interactions disrupt HSP90-kinase client homeostasis, leading to inappropriate folding of JAK proteins, loss of their activities, and disruption of JAK/STAT signalling [129].

ZIKV NS5 was also reported to bind and induce STAT2 degradation [58]. The structure of the ZIKV NS5-hSTAT2 complex revealed a similar domain orientation and binding mechanism to DENV NS5 [79,108]. However, unlike DENV, ZIKV NS5-mediated STAT2 degradation did not require post-translational proteolytic processing, and the first 10 residues of ZIKV NS5 were not crucial [131]. This was consistent with the observation that ZIKV NS5-mediated STAT2 degradation did not require host UBR4 nor follow the N-end rule; therefore, the precise E3 ubiquitin ligase utilized by ZIKV NS5 remains unknown [58]. In cells expressing the individual ZIKV NS5 domains, RdRp alone did not affect STAT2 levels, whereas MTase alone was sufficient to induce STAT2 degradation but with a reduced extent compared to full-length NS5 [131]. In silico analysis identified four surface residues of NS5 (K28, K45, V335, and S749) that are predicted to interact with hSTAT2 and are only present in STAT2-interacting flaviviruses (DENV and ZIKV) [132]. Substitution mutations of these NS5 residues attenuated ZIKV replication and reduced the efficiency of STAT2 antagonism, but mutation of K28 had the most pronounced effect on STAT2 degradation [133]. The SUMO modification of ZIKV NS5 was shown to be important for IFN inhibition, as NS5 with SUMO-interacting motif (SIM) mutations fail to inhibit Type-I IFN signalling [134]. Conde et al. (2020) demonstrated that SUMO-modified ZIKV NS5 was important for the regulation of IFN/STAT responses through disruption of the promyelocytic leukemic (PML) protein and its partner protein SUMO-1. PML nuclear bodies positively regulate antiviral responses by promoting the transcription of ISGs and the accumulation and stability of STAT1/2 [135], suggesting that ZIKV NS5-mediated STAT2 degradation may be related to the disruption of PML nuclear functions. Unlike DENV, nuclear localization of ZIKV NS5 was associated with the inhibition of type I IFN signalling but through a STAT2 degradation-independent mechanism [136].

Through the impediment of STAT2 by NS5, STAT1 homodimerization is favoured during ZIKV infections, leading to selective activation of IFN-γ signalling and transcription of genes under GAS regulation [137]. While ZIKV NS5 does not directly interact with STAT1 nor affect total STAT1 levels, it can inhibit STAT1 phosphorylation [28,58,138]. The underlying mechanism is unclear but may be linked to NS5-mediated disruption of HSP90 and downstream JAK1/2 and TYK2 activity [28,129]. The ZIKV Subgenomic Flaviviral RNA (sfRNA) was recently revealed as an additional requirement for NS5-mediated inhibition of STAT1 phosphorylation [28]. ZIKV sfRNA was posited to bind and stabilize NS5, leading to decreased proteasomal degradation of NS5 and sufficient accumulation required for the efficient suppression of IFN signalling [28].

The Spondweni virus (SPOV), identified as the closest known relative to ZIKV, encodes NS5 with approximately 77% amino acid similarity to ZIKV NS5 [58,117]. However, SPOV NS5 exhibits weak binding to STAT2 and does not induce degradation, inhibit its phosphorylation, or affect nuclear localization [58]. It remains unclear how ZIKV NS5-mediated IFN antagonism closely resembles the more distantly related DENV NS5 instead of SPOV.

WNV NS5 suppresses IFN signalling by downregulating IFNAR1, not through direct interaction with the receptor [139], but potentially mediated through interactions with the host protein prolidase (PEPD) [140]. PEPD is a dipeptidase important for the maturation and accumulation of IFNAR1, potentially facilitating its trafficking through the ER-Golgi network, as knockdown of PEPD alters the glycosylation and maturation of IFNAR1 [140]. The closely related West Nile Viruses, New York 99 strain (NY99) and Kunjin Virus (KUNV), both can antagonize the host interferon response. Stably replicating KUNV and WNV NY99 replicons downregulate STAT1 expression and inhibit phosphorylation of STAT1/2 and their translocation into the nucleus [141]. However, KUNV is a naturally attenuated subtype of WNV with a reduced ability to inhibit IFN signalling, owing to differences within NS5 [142]. A single residue within the RdRp (aa. 653) was responsible for the attenuation of KUNV NS5, as mutation of this residue to the corresponding residue found in NY99 (S653F) conferred KUNV NS5 the ability to inhibit pSTAT1 accumulation to comparable levels to that of NY99 [142]. Conversely, the introduction of F653S to NY99 NS5 attenuated its inhibition of STAT1 phosphorylation [142].

Tick-borne encephalitis (TBEV) and Langat viruses (LGTV) are closely related viruses; both exhibit NS5-mediated suppression of IFN signalling [143,144]. LGTV NS5 inhibits IFN signalling through interactions with the IFN receptor complex, potentially involving host factors that direct NS5 to the plasma membrane [143]. Werme et al. (2008) demonstrated that TBEV NS5 interacts with membrane protein scribble to inhibit JAK-STAT signalling [145]. Mapping the NS5 residues important for IFN antagonism revealed a high degree of similarity between LGTV and WNV [142,144]. Furthermore, both LGTV and TBEV NS5 can also bind PEPD to downregulate IFNAR1 [140]. Thus, there may be a conservation of strategies between tick-borne flaviviruses and WNV to inhibit IFN signalling [117].

These studies collectively demonstrated the importance of NS5-mediated inhibition of IFN signalling on viral replication, both in vitro and in vivo. The introduction of loss-of-function mutations in NS5 may strongly impede the ability of flaviviruses to evade innate immune responses, providing opportunities for the generation of novel live attenuated vaccines or further enhancing the safety of existing flaviviral vaccines. However, due to the highly diverse NS5-mediated evasion strategies among flaviviruses, careful considerations should be taken when identifying specific residues to target.

### 3.3. Nuclear Localization of NS5: A Potential Mechanism for Disruption

Although flaviviral RNA replication occurs within the cytoplasm, NS5 is also highly localized to the nucleus, where its functions remain enigmatic. The nuclear translocation of NS5 has been observed in various flaviviruses, including ZIKV, DENV, YFV, and JEV [146]. Multimerization and the nuclear localization of ZIKV NS5 were crucial for causing ciliopathy and the premature differentiation of neural progenitor cells [147]. Given the large molecular weight of NS5, it is likely translocated through the nuclear pore complex via active transport. Two highly conserved nuclear localization signals (NLS) were identified within (residues 320–368 and 369–405) DENV NS5, functioning through importin α/β (IMPα/β) transport [148]. However, serotype-specific differences in nuclear localization were observed with DENV NS5, with DENV2 and DENV4 NS5 predominantly located in the nucleus and cytoplasm, respectively [149]. This may be attributed to a novel monopartite NLS with the C-terminal 18 residues of DENV2 NS5, which are poorly conserved within DENV4 NS5 [150,151]. Interestingly, DENV4 NS5 is predominantly nuclear during an infection but cytoplasmic upon expression by transfection, suggesting that additional factors may regulate NS5 subcellular localization [151]. In ZIKV NS5, residues 370–406 and 11–90 were responsible for nuclear localization [136]. Incorporating mutations within the NLSs of DENV, ZIKV, and WNV NS5 has been shown to result in viruses with reduced replication and fitness [82,148,152]. Furthermore, treatment with IMPα/β1 inhibitors, Ivermectin and N-(4-hydroxyphenyl) retinamide (4-HPR), was shown to reduce ZIKV and DENV NS5 nuclear accumulation and viral replication [153,154,155]. Treatment with 4-HPR was also able to provide in vivo protection against lethal dengue infections in mice models [154]. Along with its established safety profile [156,157,158], 4-HPR demonstrates great promise as a therapeutic against dengue. The mutagenesis of the ZIKV NS5 NLS or treatment with IMPα/β1 inhibitors markedly decreased intracellular levels of NS5, suggesting that nuclear translocation may protect NS5 from proteolytic degradation in the cytoplasm [152]. The nuclear localization of NS5 may also function to dysregulate host gene transcription. The investigation of the effect of KUNV NS5 nuclear localization on the host transcriptome revealed the downregulation of genes involved in innate immune responses, response to cytokines, and complement activation [159]. The nuclear accumulation of ZIKV NS5 was important for suppressing IRF3 activation and type I IFN production [136]. DENV NS5 nuclear accumulation was associated with decreased induction of the antiviral chemokine interleukin-8 (IL-8) [160]. De Maio et al. (2016) demonstrated that nuclear DENV NS5 interacts with core components of the cellular splicing machinery to reduce mRNA splicing efficiency and alter splicing patterns of antiviral factors [161]. Similarly, ZIKV NS5 promotes nucleocytoplasmic trafficking and depletion of the host serine/arginine-rich splicing factor (SC35) [162]. One study investigating the interactomes of ZIKV and JEV NS5 identified significant enrichment of spliceosomal components and spliceosome-associated proteins (SAPs) [163], suggesting that NS5-targeting of the splicing machinery may be a conserved strategy of flaviviruses to dysregulate antiviral gene expression. During ZIKV infection, NS5 co-localizes with and sequesters IMPα to form spherical shell-like nuclear bodies, which are associated with the upregulation of pro-inflammatory genes [164]. Within the nucleus, ZIKV NS5 can also bind chromatin DNA to block transcriptional elongation of target genes or bind centrosomal proteins for mitotic dysfunction [165,166].

To summarize, the nuclear localization of NS5 may be a promising avenue for the development of novel therapeutics, as nuclear transport inhibitors may hinder viral replication through disruption of NS5 nuclear functions or enhancing proteasomal degradation of NS5. Furthermore, these inhibitors have the potential for broad-spectrum antiviral activity against multiple flaviviruses, as their underlying nuclear transport mechanism may be similar. However, further understanding of the precise role of NS5 nuclear localization will be crucial.

## 4. The Flavivirus NS5 as a Target for Development of Novel Vaccines and Therapeutics

### 4.1. Targeting the NS5 for Design of Live Attenuated Vaccine Candidates

NS5 performs various crucial roles required for viral replication, such as RNA methylation, replication, and interference of IFN signalling. The incorporation of mutations into NS5 is a viable strategy for the design of live attenuated vaccines or enhancing the safety profile of existing vaccines, as mutations may confer greater degrees of viral attenuation due to residues having multiple functions. A single amino acid substitution at residue 137 of the YFV-17D vaccine virus NS5 was shown to be attenuating, resulting in a viral isolate that was uniquely attenuated in mice following intracerebral inoculation [167]. Hanley et al. (2002) incorporated a series of charge-to-alanine mutations into DENV4 NS5 to generate a panel of live attenuated vaccine candidates and demonstrated that virus attenuation can be further modified by combining two pairs of charge-to-alanine mutations [168]. The mild reactogenicity of the live attenuated DENV4 vaccine candidate, rDEN4Δ30 (30 nt deletion within 3′UTR), was then reduced through paired charge-to-alanine mutagenesis of NS5 residues at positions 200 and 201 within the MTase domain [169]. The resulting rDEN4Δ30-200,201 vaccine candidate displayed restricted replication in human liver cells and was highly attenuated in rhesus macaques in comparison to DENV4 WT and rDEN4Δ30, demonstrating a 250- and 40-fold reduction in peak viraemia, respectively [168,169,170]. Furthermore, the mutations at residues 200 and 201 each independently contributed to the attenuation of rDEN4Δ30 [171], suggesting that such paired mutations are likely to be phenotypically stable. To develop a live attenuated virus vaccine against the St. Louis encephalitis virus (SLEV), a flavivirus of the JEV serocomplex, a SLEV/DENV4 chimeric virus was developed [172]. The addition of paired charge-to-alanine mutations in DENV4 NS5 at residues 436 and 437 resulted in viruses with a 400,000-fold reduction in peak viral titres compared to SLEV in the mouse brain but not more attenuated than SLEV/DENV4 in rhesus monkeys [172]. In contrast, the incorporation of paired mutations at residues 654 and 655 of DENV4 NS5 was highly attenuating upon infection of both mice brain and rhesus monkey, and immunization with this virus was able to confer complete protection against SLEV challenge in monkeys [172]. The chimeric TBEV/DEN4Δ30 live attenuated vaccine was developed to provide a safe and efficacious vaccine against tick-borne encephalitis (TBE), yet it still raises safety concerns due to high levels of neurovirulence in animal models [173]. To improve the safety profile of the chimeric TBEV/DEN4 vaccine, Engel et al. (2010) similarly incorporated paired NS5 mutations at positions 654 and 655 and a single mutation in the E protein. The resulting TBEV vaccine candidate was up to 487-fold less neurovirulent, replicated poorly within the suckling mouse brain, and was not neuroinvasive in immunodeficient mice [174].

Flaviviruses bearing mutations within the MTase catalytic K-D-K-E tetrad are severely attenuated due to their inability to perform 2′-O methylation of the 5′ cap, which shields viral RNA from effector functions of interferon-induced protein with tetratricopeptide repeat (IFIT) proteins [175]. Zust et al. (2013) generated DENV1/2 strains bearing mutations within the K-D-K-E catalytic tetrad (E216A in DENV1 and E217A in DENV2) of the MTase domain as live attenuated vaccine candidates [176]. The infection of mice and rhesus monkeys with DENV 2′-O-MTase mutants demonstrated severe attenuation of viruses, increased sensitivity to IFN-β treatment, and protection against subsequent DENV challenge [176]. The same approach was opted by Li. et al. (2013) for the development of a JEV vaccine, in which the JEV 2′-O-MTase mutant was attenuated, demonstrated increased sensitivity to IFN-α, and induced robust humoral and cellular immune responses in mice [177]. The role of 2′-O methylation is likely conserved across many other flaviviruses, suggesting that K-D-K-E tetrad mutations may also be a viable strategy to generate attenuated vaccines for other flaviviruses [175,178].

### 4.2. The NS5 Elicit Potent T-Cell Immune Responses

During a flaviviral infection, NS5 proteins expressed in large excess are targets for proteasomal degradation by the host ubiquitin–proteasome system, generating peptides that are presented on the cell surface in Class I MHC complexes and presentation of NS5 epitopes to T-cell populations [179,180]. The activation and clonal expansion of these T-cells will establish an effective immune response, and the long-term persistence of virus-specific memory T-cells will protect against subsequent infections. This was evident in primary infections of Indian rhesus macaques, in which robust CD4+ and CD8+ T-cell responses were induced against NS5 epitopes, followed by sustained IFN-γ responses against NS5 3 weeks after viral clearance [47]. It was also shown that human CD8+ T-cells mainly target NS3 and NS5 during a natural DENV infection, highlighting the immunodominance of these NS proteins [181]. However, the pattern of immunodominant T-cell epitopes was different between DENV serotypes during natural infections, as DENV1, DENV2, and DENV4 mainly elicit CD8+ T-cell responses against NS proteins, NS3 and NS5, whereas DENV3-specific responses are directed towards structural proteins [182]. In subjects receiving the live-attenuated tetravalent dengue vaccines, TDV (DENVax) and TV003, NS3 and NS5 were identified as the main targets of CD8+ T-cell responses [46,48]. Furthermore, NS5 epitopes targeted after vaccination were highly conserved across DENV field isolate serotypes, and immunological patterns were similar to those observed in natural infections [46,182]. Bischof et al. (2017) developed a recombinant rhesus monkey rhadinovirus (RRV) expressing the DENV2 E and NS5 proteins. In immunized rhesus macaques, the recombinant RRV was able to generate neutralizing titres of antibodies, NS5-specific CD8+ T-cell responses, and provide moderate protection against DENV2 challenge [183]. However, T-cell frequencies were lower than unvaccinated but infected macaques, indicating that immunization with additional NS proteins may be beneficial [183]. Roth et al. (2019) developed a T-cell-based vaccine encoding for a minimal DENV1 antigen comprised of highly conserved and antigenic T-cell epitopes derived from NS3, NS4B, and NS5. The immunization of HLA transgenic mice with the DENV1-NS Poly-Epitope induced strong T-cell responses and protective immune memory against the DENV1 challenge without the need for neutralizing antibody activity [184].

No subunit vaccine for flaviviruses is currently commercially available, although several E protein-based vaccines are currently in clinical trials [185]. Alves et al. (2016) investigated the potential of NS5 as a subunit vaccine antigen [186]. Mice immunized with purified recombinant DENV2 NS5 expressed in *E. coli*-induced serum NS5-specific IgG and IFN-γ and TNF-α secreting T-cells [186]. Furthermore, immunization with NS5 alone induced partial protection against the DENV2 challenge, with 60% and 80% protection against the JHA1 and NGC strains, respectively [186].

Schouest et al. (2021) observed cross-reactive T-cell responses against ZIKV from DENV-exposed PBMC samples, with non-structural proteins representing 76% of epitopes and NS5 being the most dominantly targeted and recognized antigen [187]. Vaccination with TV003 vaccines can also induce cross-reactive CD8+ T responses across DENV serotypes and ZIKV [188]. This suggests that prior DENV infection or vaccination and pre-existing cellular immunity may confer protection against subsequent DENV or ZIKV infection. Indeed, cross-reactive CD8+ T-cell responses in DENV-immune mice were shown to provide enhanced protection against subsequent ZIKV infection [189]. Being the most conserved NS protein, NS5 has the potential to induce cross-protective immunity across flaviviruses and among their serotypes. The phylogenetic analysis of DENV and ZIKV NS5 amino acid sequences has identified up to 19 epitopes that are 100% conserved among ZIKV lineages and DENV serotypes [190], and targeting these epitopes is a promising strategy for cross-protective vaccine design.

Collectively, these studies further corroborate the importance of T-cell-based immune responses targeting NS5 and other NS proteins to confer protective immunity and provide a rationale for future vaccines that rely on immunodominant NS5 epitopes and T-cell activation.

### 4.3. Targeting the NS5 for Proteasomal Degradation as a Novel Strategy for Therapeutics

Given the importance of the ubiquitin–proteasome system in the presentation of viral peptides in class I MHC complexes to T-cells, novel strategies to direct NS5 towards proteasomal degradation may be a viable strategy to enhance viral epitope presentation. Proteolysis-targeting chimeric (PROTAC)-based vaccines are designed to direct viral proteins for proteasomal degradation through the addition of a proteasome-targeting domain (PTD) [191]. Flavivirus vaccines utilizing PROTAC technology may be a viable strategy to direct NS5 proteins towards the ubiquitin–proteasome system to enhance NS5 epitope presentation and T-cell-based immunity. This can also result in decreased NS5 levels and a corresponding loss of its functions in viral replication and antagonism of IFN signalling, thus conferring greater degrees of attenuation and safety compared to existing vaccines. However, PROTAC-based vaccines were only demonstrated for the Influenza A virus, and further research is still required to determine if PROTAC technology can apply to flaviviruses. RNA-PROTACs, which are RNA molecules with a conjugated E3-recruiting peptide, may be an alternative strategy to direct RNA-binding proteins, such as NS5, towards proteasomal degradation. Other strategies to enhance NS5 proteasomal degradation include the inhibition of NS5 nuclear localization, which may function to protect NS5 from cytoplasmic degradation [152] or interference of NS5 interaction with the subgenomic flaviviral RNA (sfRNA), which was recently shown to be a viral factor required for the stabilization and cytosolic accumulation of ZIKV NS5 [28]. The disruption of the sfRNA-NS5 interaction through mutations of their interacting sites or the generation of viruses with mutations within the 3′UTR to abolish sfRNA generation may be novel strategies to direct NS5 towards proteasomal degradation. Small molecule inhibitors that disrupt NS5-sfRNA interactions may be a novel strategy for the development of flavivirus-specific antiviral treatments. Several live attenuated vaccine candidates contain deletions within the 3′UTR (e.g., rDEN4Δ30, ZIKV-3′UTR-Δ10) and are sfRNA-deficient viruses [192,193]. The interference of the NS5-sfRNA interaction in sfRNA-deficient vaccines may contribute to their attenuation, although their underlying attenuating mechanism is still unclear and warrants further studies.

### 4.4. The NS5 Is a Prime Target for Development of Small Molecule Antiviral Inhibitors

The crucial functions of NS5 in flavivirus replication and its highly conserved structure make it an attractive target for inhibition and the development of broad-spectrum small molecule inhibitors. The SAM binding site of the MTase domain is a prime target for drug design and screening. However, the design of flavivirus MTase inhibitors presents challenges, as human methyltransferases have similar core domains and use SAM as a cofactor [194]. For instance, sinefungin (SIN) displayed broad-spectrum inhibition against WNV, DENV, and YFV, but chemical similarities between SIN and SAM raise concerns about cytotoxicity [195]. The introduction of modifications in SAM/SAH can generate analogues with selective activity against the flaviviral MTase and reduced cytotoxicity [196]. A conserved hydrophobic pocket adjacent to the SAM/SAH binding site unique to NS5 and absent in human MTases can also be exploited to develop highly selective inhibitors [197]. Jain et al. (2017) developed a novel SAM analogue (MS2042) with a 4-fluorophenyl moiety that exploited binding to the conserved hydrophobic pocket in ZIKV MTase [198]. Lim et al. evaluated a series of SAH derivatives with modifications at the N6 position of the adenine base to identify small molecule inhibitors that specifically target DENV MTase [199]. Brecher et al. (2015) performed virtual screening to identify two compounds, NSC 12155 and NSC 125910, that inhibit MTase function by competitively inhibiting SAM binding to the MTase cofactor pocket [195]. The guanylyltransferase activity of MTase is also a potential mechanism for inhibition, as Stahla-Beek et al. (2012) identified a family of compounds, 2-thioxothiazolidin-4-ones, that can strongly inhibit GTP binding [200]. Thames et al. (2020) developed novel flexible nucleoside analogues binding to the GTP pocket of DENV and ZIKV MTase to inhibit virus replication [201]. An alternative strategy to increase the specificity of MTase inhibitors is to target non-catalytic sites (allosteric sites) [202]. Benmansour et al. (2017) performed fragment-based screening to identify allosteric inhibitors against structurally conserved sites of the MTase [203], and Coutard et al. (2017) demonstrated that the identified allosteric inhibitors were similarly effective against ZIKV MTase [202].

The viral RdRp plays an important role in the life cycle of RNA viruses such as Hepatitis C virus (HCV), influenza viruses, coronaviruses, and flaviviruses [204]. Inhibitors of RdRp activity (i.e., Sofosbuvir) have already been demonstrated to be highly effective against HCV infections [205]. The core structure of the RdRp (fingers, palm, and thumb subdomains) is highly conserved across RNA viruses, suggesting that RdRp inhibitors may demonstrate broad-spectrum antiviral activity [206].

Current RdRp inhibitors include nucleoside analogue inhibitors (NI), which compete with nucleotides and terminate RNA chain elongation, and non-nucleoside analogue inhibitors (NNI), which bind to catalytic or allosteric sites and inhibit RdRp activity. Several novel nucleoside analogues have been identified against multiple flaviviruses [207,208,209], and previously identified drugs have also been repurposed against flaviviruses [210]. The nucleoside inhibitor Sofosbuvir was also shown to inhibit the replication of various flaviviruses (DENV, WNV, ZIKV, and YFV) in vitro and has great potential to be repurposed for flaviviral infections [211,212,213,214]. Niyomrattanakit et al. (2010) demonstrate that N-sulfonylanthranilic acid derivatives act as NNIs of DENV NS5 by binding to the RNA template tunnel and hindering RdRp activity [215]. Tarantino et al. (2016) identified a pyridobenzothiazole compound, HeE1-2Tyr, that inhibited a panel of flaviviral RdRps by binding a distinct site between the fingers domain and the priming loop [216]. Lim et al. (2016) identified inhibitors binding to an interface between the palm and thumb subdomain (termed the ‘N’ pocket), which hinders conformational changes of the RdRp during its transition to RNA elongation [217]. The first-in-class antibiotic fidaxomicin may be repurposed as an antiviral agent for ZIKV infections, as it was reported to inhibit RdRp activity and inhibit ZIKV replication both in vitro and in vivo. Lin et al. (2019) demonstrated that the antifungal agent, 10-undecenoic acid zinc salt (UA), could inhibit ZIKV RdRp by binding to the catalytic site [218].

The inhibition of flaviviral protein–protein interactions (PPI) to disrupt the assembly of NS proteins within the replication complex represents a novel avenue for drug development. Celegato et al. (2023) demonstrated that inhibitors of NS5-NS3 interaction inhibit the replication of multiple mosquito-borne flaviviruses and also proved efficacious in a mouse model of DENV infection [219]. Cannalire et al. (2020) demonstrated that pyridobenzothiazolones compounds inhibit RdRp activity, NS5-NS3 interaction, and NS5-3′UTR interactions [220]. The importance of NS5-RNA interactions has been demonstrated across flaviviruses [17,18,28]; however, it remains a largely unexplored avenue in flaviviral therapeutic development and warrants further studies.

## 5. Conclusions

Flaviviruses represent an emerging and significant global health threat. However, there are very few vaccines that are commercially available for flaviviruses, restricted to YFV, JEV, TBEV, and DENV [185]. There is an urgent need for vaccines against emerging threats such as ZIKV, WNV, and many other lesser-known flaviviruses with potential for epidemics [1]. The mechanism underlying protective immunity against flaviviral infections remains poorly understood. The notion that neutralizing antibodies are the main effector of protection against flaviviral infection has been continually challenged by subsequent studies reinforcing the importance of T-cell-based responses against NS proteins. The development of vaccines targeting NS proteins abolishes the risk of individuals developing severe flaviviral disease due to ADE, with NS5 being a notably promising vaccine target due to its ability to elicit an immunodominant immune response [181]. Furthermore, it is the most conserved NS protein and has the potential to elicit cross-protective immunity [221]. Further studies to identify immunodominant and conserved epitopes will facilitate the development of novel T-cell-based vaccines. Furthermore, the flavivirus NS5 performs crucial functions in viral replication and antagonism of host immune responses, as detailed by studies in this review, providing opportunities for the development of novel therapeutics.

## Figures and Tables

**Table 1:** Flavivirus vaccines that are licensed or are undergoing clinical trials.

Flavivirus	Vaccine Candidate	Manufacturer	Vaccine Technology	Current Progress
DENV	Dengvaxia (CYD_TDV)	Sanofi Pasteur	Live chimeric virus	Licensed
	Qdenga (TAK-003)	Takeda	Live chimeric virus	Licensed
YFV	YF-17DD	Bio-Manguinhos/Fiocruz	Live attenuated virus	Licensed
	YF-VAX/Stamaril	Sanofi Pasteur	Live attenuated virus	Licensed
	YFV-17D-213	Federal State Unitary Enterprise of Chumakov Institute	Live attenuated virus	Licensed
	17D-204	Institut Pasteur Dakar (Senegal)	Live attenuated virus	Licensed
JEV	IXIARO/JESPECT/JEEV (JE-VC)	Valneva	Inactivated whole virus	Licensed
	IMOJEV (JE-CV)	Acambis/Sanofi Pastuer	Live attenuated virus	Licensed
	SA 14-14-2	BBIL, CDIBP, Chengdu Institute of Biological Product	Live attenuated virus	Licensed
	JEVAC	Liaoning Cheng Da Biotechnology Co., Ltd.	Inactivated whole virus	Licensed
	JEBIK^®^V	Biken	Inactivated whole virus	Licensed
	ENCEVAC	KM-Biologics, Kaketsuken; Boryung	Inactivated whole virus	Licensed
	JEVAX	VABIOTECH	Live attenuated virus	Licensed
	JEV-GCC	Green Cross Corp	Inactivated whole virus	Licensed
	JENVAC	Bharat Biotech	Inactivated whole virus	Licensed
TBEV	TBE-Moscow	Chumakov Institute of Poliomyelitis and Viral Encephalitides	Inactivated whole virus	Licensed
	EnceVir	Microgen	Inactivated whole virus	Licensed
	FSME-Immun/Tico Vac	Baxter, Pfizer	Inactivated whole virus	Licensed
	Tick-E-Vac	Chumakov FSC R&D IBP RAS	Inactivated whole virus	Licensed
	SenTaiBao	Changchun Institute of Biological Products	Inactivated whole virus	Licensed
	Encepur	Bavarian Nordic	Inactivated whole virus	Licensed
WNV	WN/DEN4-3′Δ30	NIAID	Live chimeric virus	In Trials
	HydroVax-001	Najit Technologies	Inactivated whole virus	In Trials
	WN-80E	Hawaii Biotech	Protein subunit	In Trials
	VRC-WNVDNA020-00-VP	NIAID, NIH, DVBD, CDC	Genomic (DNA)	In Trials
	VRC-WNVDNA017-00-VP	NIAID, NIH, DVBD, CDC	Genomic (DNA)	In Trials
	ChimeriVax-WN02	Sanofi Pasteur	Live chimeric virus	In Trials
DENV	TetraVax-DV—TV-003	NIAID	Live attenuated virus	In Trials
	TetraVax-DV—TV-005	NIAID	Live attenuated virus	In Trials
	TetraVax-DV—V181	Merck & Co., Instituto Butantan, and Medigen Vaccine Biologics	Protein subunit	In Trials
	TDEN	WRAIR and GSK	Live attenuated virus	In Trials
	TDEN-F17	WRAIR and GSK	Live attenuated virus	In Trials
	TDEN-F19	WRAIR and GSK	Live attenuated virus	In Trials
	TVDV	WRAIR	Genomic (DNA)	In Trials
	CYD-1,2,3,4/VDV-2	Sanofi	Live chimeric virus	In Trials
	Dengusiil	Serum Institute of India Pvt. Ltd.	Live attenuated virus	In Trials
	DSV4	International Centre for Genetic Engineering and Biotechnology	Protein subunit	In Trials
	E80-mRNA	CAS laboratory of Molecular Virology and Immunology, Institute Pasteur of Shanghai	mRNA	In Trials
	TDENV-PIV	WRAIR and GSK	Inactivated whole virus	In Trials
	TDENV-LAV	WRAIR and GSK	Live attenuated virus	In Trials
	PepGNP-Dengue	Emergex Vaccines	Nanoparticle antigen delivery	In Trials
	rDEN1∆30	NIAID	Live attenuated virus	In Trials
	rDEN2/4Δ30	NIAID	Live chimeric virus	In Trials
	rDEN2/4Δ30(ME)	NIAID	Live chimeric virus	In Trials
	rDEN3∆30	NIAID	Live attenuated virus	In Trials
	rDEN3/4Δ30(ME)	NIAID	Live chimeric virus	In Trials
	rDEN3Δ30/31-7164	NIAID	Live attenuated virus	In Trials
	rDEN3-3′D4Δ30	NIAID	Live chimeric virus	In Trials
	rDEN4Δ30	NIAID	Live attenuated virus	In Trials
	rDEN4Δ30-200,201	NIAID	Live attenuated virus	In Trials
	rDEN4Δ30-4995	NIAID	Live attenuated virus	In Trials
ZIKV	ZPIV	NIAID/WRAIR/BIDMC	Inactivated whole virus	In Trials
	rZIKV/D4Δ30-713	NIAID	Live chimeric virus	In Trials
	mRNA-1893	Moderna	mRNA	In Trials
	MV-ZIKA	Themis Bioscience GmbH	Live chimeric virus	In Trials
	MV-ZIKA-RSP	Themis Bioscience GmbH	Viral vector-based	In Trials
	VRC-ZKADNA085-00-VP	NIAID/VRC	Genomic (DNA)	In Trials
	VRC-ZKADNA090-00-VP	NIAID/VRC	Genomic (DNA)	In Trials
	VLA1601	Valneva	Inactivated whole virus	In Trials
	ChAdOx1 Zika	University of Oxford	Viral vector-based	In Trials
	BBV121	Bharat Biotech	Inactivated whole virus	In Trials
	GLS-5700	Inovio Pharmaceutical	Genomic (DNA)	In Trials
	PIZV (TAK-426)	Takeda Pharmaceuticals	Inactivated whole virus	In Trials
	ChimeriVax-Zika (CYZ)	Sanofi	Live chimeric virus	In Trials
YFV	XRX-001	Xcellerex	Inactivated whole virus	In Trials
	SII YFV	Serum Institute of India Pvt. Ltd.	Live attenuated virus	In Trials
	vYF	Sanofi Pasteur	Live attenuated virus	In Trials
JEV	JECEVAX	VABIOTECH	Inactivated whole virus	In Trials
